# Natural Pumice-Filled Flexible Polyurethane Composites: Effect of Filler Content on Mechanical Performance and Cyclic Durability

**DOI:** 10.3390/polym18182270

**Published:** 2026-09-17

**Authors:** Omer Uctu, Mehmet Ipekoglu, Onder Albayrak

**Affiliations:** 1 Department of Mechanical Engineering, Institute of Science, Mersin University, Mersin 33343, Türkiye; 2 Department of Textile, Clothing, Footwear and Leather, Naci Topcuoglu Vocational School, Gaziantep University, Gaziantep 27600, Türkiye; omeructu@gantep.edu.tr; 3 Department of Mechanical Engineering, Faculty of Engineering, Turkish-German University, Istanbul 34820, Türkiye; 4 Department of Mechanical Engineering, Faculty of Engineering, Mersin University, Mersin 33343, Türkiye

**Keywords:** polyurethane composites, pumice, filler content, mechanical performance, abrasion resistance, cyclic flexural durability

## Abstract

This study investigates the effect of natural pumice filler content on the physical and mechanical performance of flexible polyester-based polyurethane (PU) composites. Composites containing 0, 2, 5, 10, and 20 wt.% pumice were produced using a constant PU matrix formulation and identical processing conditions. Microstructural and structural characteristics were examined using scanning electron microscopy (SEM), energy-dispersive X-ray spectroscopy (EDX), and Fourier transform infrared spectroscopy (FTIR), while density, water absorption, tensile behavior, abrasion resistance, and cyclic flexural durability were evaluated. Increasing pumice content resulted in greater local microstructural heterogeneity and increased density and water absorption. Tensile strength exhibited a non-linear numerical response to filler content, with the highest mean value obtained at 5 wt.% pumice, although the differences among formulations were not statistically significant. In contrast, elongation decreased significantly overall with increasing filler content, while mean abrasion mass loss progressively increased with filler loading. Cyclic flexural testing revealed that the unfilled PU and 2 wt.% composite completed 30,000 cycles without measurable crack propagation at 20 and 0 °C, whereas higher filler contents exhibited severe crack growth or complete fracture. At −20 °C, all formulations fractured completely. Overall, pumice content produced a property-dependent response, revealing a trade-off among tensile response, deformability, abrasion resistance, and cyclic durability. These findings provide a basis for tailoring flexible PU composites for applications involving repeated deformation and mechanical contact, including footwear and cushioning components.

## 1. Introduction

Flexible polyurethane (PU) materials are widely used in engineering applications requiring low density, high deformability, energy absorption, and resistance to repeated mechanical loading, including cushioning systems, automotive components, sports equipment, and footwear applications [1,2,3,4,5]. In such applications, material performance cannot be assessed solely through static strength. The ability to retain deformability, resist abrasive wear, and withstand repeated loading under different service temperatures is also critical, particularly for flexible components subjected to continuous mechanical contact and cyclic deformation [1,2].

The incorporation of inorganic and naturally derived fillers is a widely used approach for tailoring the physical and mechanical performance of PU composites. Calcium carbonate, silica, talc, clay minerals, natural fibers, and recycled materials have been investigated for this purpose [6,7,8,9]. Depending on filler type, content, dispersion, and filler–matrix interactions, the dispersed phase may contribute to load transfer and modify the tensile response of the polymer matrix [1,10,11]. However, increasing filler content does not necessarily produce a continuous improvement in mechanical performance. Excessive filler loading may restrict matrix deformation and promote local heterogeneity or particle agglomeration, resulting in reduced elongation and deterioration of mechanical properties [1,12,13]. Consequently, filler content represents a critical design parameter for achieving an appropriate balance between reinforcement and deformation capability.

The effects of fillers on flexible PU systems depend strongly on filler type, particle size, loading, surface characteristics, and filler–matrix interactions. For mineral-filled flexible PU foams, Javni et al. [14] showed that microfillers increased density, hardness, compression strength, and compression set but reduced elongation at break, whereas hydrophilic nanosilica improved hardness, compression strength, rebound resilience, tensile strength, and tear strength. Latinwo et al. [15] similarly demonstrated a strong particle-size and concentration dependence for calcite (CaCO_3_) and dolomite fillers over a broad loading range of 0–40 wt.%; nano-sized fillers produced more pronounced improvements in indentation hardness, whereas tensile strength and elongation decreased as filler loading increased. In clay-filled flexible PU foams, Ok et al. [16] reported improvements in thermal stability together with changes in cellular morphology and mechanical properties, with the most favorable overall response obtained at an intermediate nanoclay content and deterioration occurring at higher loading. A comparable concentration-dependent response was observed by Piszczyk et al. [17] for thermally reduced graphene oxide, for which 0.50 wt.% increased foam rigidity, glass-transition temperature, and thermal stability, while further addition led to deterioration of the physicochemical properties.

Similar filler-dependent behavior has also been reported for non-foamed PU systems. Zhang et al. [18] investigated kaolin-filled polyurethane elastomers and showed that the mechanical response depended on both filler content and kaolin treatment, with low filler loading providing improved mechanical performance relative to the unfilled matrix. Shahzamani et al. [19] investigated BaSO_4_, CaCO_3_, kaolin, and quartz fillers in cast polyurethane over a concentration range of 0–40 parts per hundred resin (phr). Their experimental results and SEM observations indicated that the formulation containing 30 phr CaCO_3_ provided the best overall performance among the investigated formulations. Natural and recycled fillers have likewise been explored as alternatives to conventional inorganic fillers in flexible PU systems [4,5], further demonstrating that filler incorporation can modify several properties simultaneously rather than producing a uniform reinforcement effect.

Taken together, these studies show that filler incorporation into PU involves a recurring trade-off between reinforcement and deformability. Improvements in hardness, stiffness, compressive response, or tensile performance may be obtained at appropriate filler contents, whereas excessive loading can reduce elongation and other mechanical properties [14,15,16,17,18,19]. The reported optimum filler level varies substantially among studies, from sub-percent concentrations for nanoscale fillers [17] to considerably higher loadings for conventional mineral fillers [15,19]. Direct quantitative comparison among these systems is therefore difficult because the PU formulations, filler chemistry and particle size, loading basis, surface treatment, cellular structure, and processing conditions differ considerably. Nevertheless, the collective results demonstrate that filler concentration cannot be considered independently of filler characteristics and PU microstructure when balancing reinforcement and flexibility.

For flexible PU composites, this balance becomes particularly important under service-related loading conditions. Tensile performance alone may not adequately represent the mechanical response of a material subjected to repeated deformation and surface contact. Cyclic flexural durability and abrasion resistance provide complementary information regarding resistance to crack propagation and material removal during service. Previous studies have shown that filler incorporation can significantly influence deformation and damage development under repeated loading, while temperature can further modify the cyclic response of flexible polymer systems [12,20]. Similarly, abrasion performance depends on the combined response of the polymer matrix, dispersed filler phase, and resulting microstructure under mechanical contact [11,21]. Physical characteristics such as density and water absorption must also be considered because changes in these properties may accompany any mechanical benefit obtained through filler incorporation [22,23].

Among naturally occurring mineral fillers, pumice is an attractive candidate because of its abundance, porous structure, relatively low density, and silicate-rich composition. Pumice has been investigated as a filler in polymer systems such as polypropylene and epoxy composites, where its incorporation has been shown to modify mechanical and physical properties [24,25]. However, the response of pumice-filled flexible PU composites cannot be directly inferred from these polymer systems or from the conventional mineral-filled PU systems discussed above because filler effects are strongly dependent on matrix characteristics, filler type, particle size, concentration, and microstructure.

Overall, the current literature shows that the effects of mineral fillers in PU systems are strongly dependent on filler characteristics, concentration, and matrix structure, with improvements in one property often accompanied by deterioration in others. Within this broader research context, the use of pumice in flexible PU composites remains comparatively underexplored, particularly with respect to the combined effects of filler content on tensile behavior, abrasion resistance, water absorption, and cyclic durability under different service temperatures.

Accordingly, the present study systematically investigates the effect of pumice filler content on the microstructure, physical characteristics, and mechanical performance of flexible polyester-based PU composites. The PU matrix formulation and processing conditions were maintained constant, while pumice content was varied from 0 to 20 wt.% using a nominal particle size of 40 µm. SEM, EDX, and FTIR analyses were employed as supporting characterization methods, whereas the performance assessment focused on density, water absorption, tensile behavior, abrasion resistance, and cyclic flexural durability at 20, 0, and −20 °C. The study aims to clarify the property-dependent response of flexible PU composites to increasing pumice content and to identify the trade-offs among tensile response, deformability, wear resistance, and cyclic durability. Although footwear midsoles provide a representative application context, the findings are intended to support the broader design of flexible PU composites subjected to repeated deformation and mechanical contact.

## 2. Materials and Methods

### 2.1. Mold Design

A laboratory-scale mold system was designed and manufactured to produce flexible PU composite specimens under controlled and reproducible processing conditions. A flat aluminum mold geometry was selected to obtain specimens with uniform thickness and to provide sufficient material for subsequent structural and mechanical characterization.

The mold was designed to withstand the internal pressure generated during the foaming and curing processes. The upper and lower mold plates were mechanically clamped using threaded bolts and fastening nuts to maintain mold closure during polymerization. Steel hinges were incorporated to facilitate opening, demolding, and repeated specimen production.

To ensure controlled and uniform processing conditions, resistive heating elements were installed on the upper and lower mold plates, while thermocouples positioned near the mold cavity were used to monitor the mold temperature. The heating system was connected to an electrical control unit to maintain the prescribed processing temperature throughout specimen preparation. Cooling channels were also incorporated into the mold design to enable controlled heat exchange when required.

The developed mold system provided consistent thermal and mechanical boundary conditions during specimen production. All composite formulations were therefore manufactured using the same mold configuration and processing conditions, minimizing process-related variability and allowing the observed differences in material performance to be primarily evaluated as a function of pumice filler content.

### 2.2. Sample Preparation

Polyester-based PU composites were prepared using a reactive PU system consisting of a polyol blend (A), an isocyanate mixture (B), and pumice powder (C) as the inorganic filler. The polyol blend contained polyester polyol (KIMPOL PE 001, Kimpur, Istanbul, Türkiye), 1,4-butanediol (BDO, BASF SE, Ludwigshafen, Germany) as a chain extender, distilled water as a chemical blowing agent, and a tertiary amine catalyst (DABCO 25 S, Evonik, Essen, Germany). The isocyanate component consisted of monomeric methylene diphenyl diisocyanate (MDI, Kimpur, Istanbul, Türkiye) combined with a linear prepolymer. The PU matrix is formed through the reaction of hydroxyl-functional polyester polyol components with MDI-based isocyanate groups, resulting in urethane linkages, while 1,4-butanediol acts as a chain extender. The commercial polyester polyol and isocyanate components are proprietary formulations rather than single, fully defined compounds; therefore, their exact chemical structures are not publicly disclosed by the manufacturers.

According to the supplier specifications, the polyester polyol had a hydroxyl value of 56.07 mg KOH/g and a functionality of approximately 2, corresponding to an OH equivalent weight of approximately 1000 g/eq. BDO had a hydroxyl value of 1240 mg KOH/g and an OH equivalent weight of approximately 45 g/eq, while water was considered a difunctional reactive species with an OH equivalent weight of 9 g/eq.

Based on the findings of our previous study on the formulation-dependent performance of the same polyester-based PU system [26], a polyol blend/isocyanate mixture mass ratio of 50/50 was selected and maintained for all formulations. This composition corresponds to an isocyanate index of 113, based on the stoichiometric calculation established in our previous study [26]. Keeping the PU matrix formulation constant allowed pumice filler content to be treated as the primary experimental variable in the present study.

Pumice powder with a nominal particle size of 40 µm (custom-produced by Nigtas, Nigde, Türkiye) was used throughout the study. An unfilled PU formulation (PU-00) was used as the reference material, while PU composites containing 2, 5, 10, and 20 wt.% pumice were prepared to systematically investigate the effect of filler content. Pumice was incorporated while maintaining the relative proportions of the polyol-based and isocyanate components at 50/50. The compositions of the investigated formulations are summarized in Table 1.

For specimen preparation, the required amount of pumice powder was first incorporated into the polyol-based component and mechanically mixed at 2200 rpm for 60 min (615.01.001, Isolab GmbH, Wertheim, Germany) to promote uniform filler dispersion. Separately, the isocyanate component was transferred into 50 mL centrifuge tubes and centrifuged at 2000 rpm for 60 s (NF048, Nuve, Ankara, Türkiye) to remove entrapped air. The filler-containing polyol mixture and the isocyanate component were subsequently preheated at 45 °C for 1 h in an oven (FN120, Nuve, Ankara, Türkiye). Immediately before reactive mixing, the preheated polyol–pumice mixture was homogenized at 2200 rpm for 8 s. The required amounts of the polyol–pumice mixture and isocyanate component were then combined and mechanically mixed at 2200 rpm for an additional 8 s.

The resulting reactive mixture was immediately poured into the aluminum mold preheated to 50 °C. The mold was mechanically closed using the bolt-and-nut clamping system described in Section 2.1 and maintained under these conditions for 300 s. After the molding period, the mold was opened, and the PU composite specimen was removed. The same preparation procedure and processing parameters were applied to all formulations to minimize manufacturing-related variability and enable the effect of pumice filler content on the microstructure and mechanical performance of the composites to be evaluated under consistent processing conditions.

### 2.3. Characterization

Prior to characterization, the PU composite specimens were conditioned according to TS EN 12222 [27] at 20 ± 2 °C and 50 ± 5% relative humidity for 24 h in a laboratory conditioning cabinet (TK120, Nuve, Ankara, Türkiye). The characterization procedure was designed primarily to evaluate the influence of pumice filler content on the microstructure, physical characteristics, and mechanical performance of the PU composites.

The microstructural characteristics of the composites were examined by scanning electron microscopy (SEM; Zeiss Gemini 300, Carl Zeiss Microscopy GmbH, Oberkochen, Germany). Prior to observation, the specimen surfaces were gold-coated using a sputter coater (SC7620, Emitech, Ashford, UK). SEM images were used to assess changes in the overall morphology and local microstructural heterogeneity of the PU composites as a function of filler content. Elemental analysis was performed using an energy-dispersive X-ray fluorescence spectrometer (Shimadzu EDX-7000, Shimadzu Corporation, Kyoto, Japan) operated with PCEDX-Navi software (Version 2.01, Shimadzu Corporation, Kyoto, Japan) to verify the presence of the inorganic filler constituents in the composites. Fourier transform infrared spectroscopy (FTIR) was performed using a spectrometer (Shimadzu IR-Affinity 1S, Shimadzu Corporation, Kyoto, Japan) equipped with a MIRacle ATR accessory. Disc-shaped specimens (16 mm in diameter and 7.5 mm in thickness) were cut from the molded samples, and the surface silicone layer was removed before analysis. Spectra were acquired after background correction and were used to compare the characteristic absorption bands of the unfilled and pumice-containing PU composites.

The densities (ρ) of three specimens were calculated from their measured masses (m) and volumes (V). Mass was determined using an analytical balance (Shimadzu ATX224, Shimadzu Corporation, Kyoto, Japan), while specimen volume was determined by water displacement based on the Archimedes principle using distilled water at approximately 23 °C.

Water absorption was determined on three samples in accordance with TS 5501 [28]. Cubic specimens (5 × 5 × 5 mm^3^) were dried and weighed to obtain the initial mass (M_0_), immersed in distilled water for 24 h, and subsequently reweighed (M_1_). Water absorption was calculated from the mass increase relative to the initial dry mass.

Abrasion resistance of the three samples was evaluated according to TS EN 12770 [29] using an abrasion tester (DIN abrasion tester HD-P307, Haida International Equipment, Dongguan, China). Cylindrical specimens with a diameter of 16 ± 0.2 mm were abraded against 60-grit abrasive paper over a sliding distance of 20 m at 42 rpm under a normal load of 7.5 ± 0.1 N. Abrasion performance was quantified from the mass loss measured before and after testing.

Tensile tests were performed on three independent specimens for each formulation using a universal testing machine with a 50 kN load cell (Shimadzu AGS-X, Shimadzu Corporation, Kyoto, Japan) at a crosshead speed of 80 mm/min in accordance with TS EN ISO 17709 [30] and TS EN 12803 [31]. Dumbbell-shaped specimens were cut from equivalent locations of the molded samples to minimize specimen-position-related variability. Tensile stress and elongation were determined to evaluate the effect of pumice content on the load-bearing capability and deformation response of the PU composites.

Cyclic flexural resistance was conducted on the three samples using a flexural testing machine (HD-E702-150B40, Haida International Equipment, Dongguan, China) in accordance with TS EN ISO 17707 [32]. An incision of 2 ± 0.1 mm was introduced at the prescribed flexing line of each specimen, and the specimens were subjected to cyclic bending at 100 cycles/min for 5 h (30,000 cycles). Tests were conducted at −20, 0, and 20 °C to assess the resistance of the composites to crack propagation under repeated deformation at different service temperatures. Following testing, the increase in incision length was measured and used as an indicator of cyclic flexural durability.

### 2.4. Statistical Analysis

Density, water absorption, tensile strength, elongation at break, and abrasion mass loss were determined using three independent specimens for each formulation (*n* = 3), and the results are presented as mean ± standard deviation (SD). The statistical significance of the effect of pumice content on each property was evaluated using one-way analysis of variance (ANOVA). For properties showing a significant overall ANOVA result, Tukey’s honestly significant difference (HSD) post hoc test was used for pairwise comparisons among formulations. Differences were considered statistically significant at *p* < 0.05.

## 3. Results and Discussion

### 3.1. Microstructural and Structural Characterization

**Figure 1:**
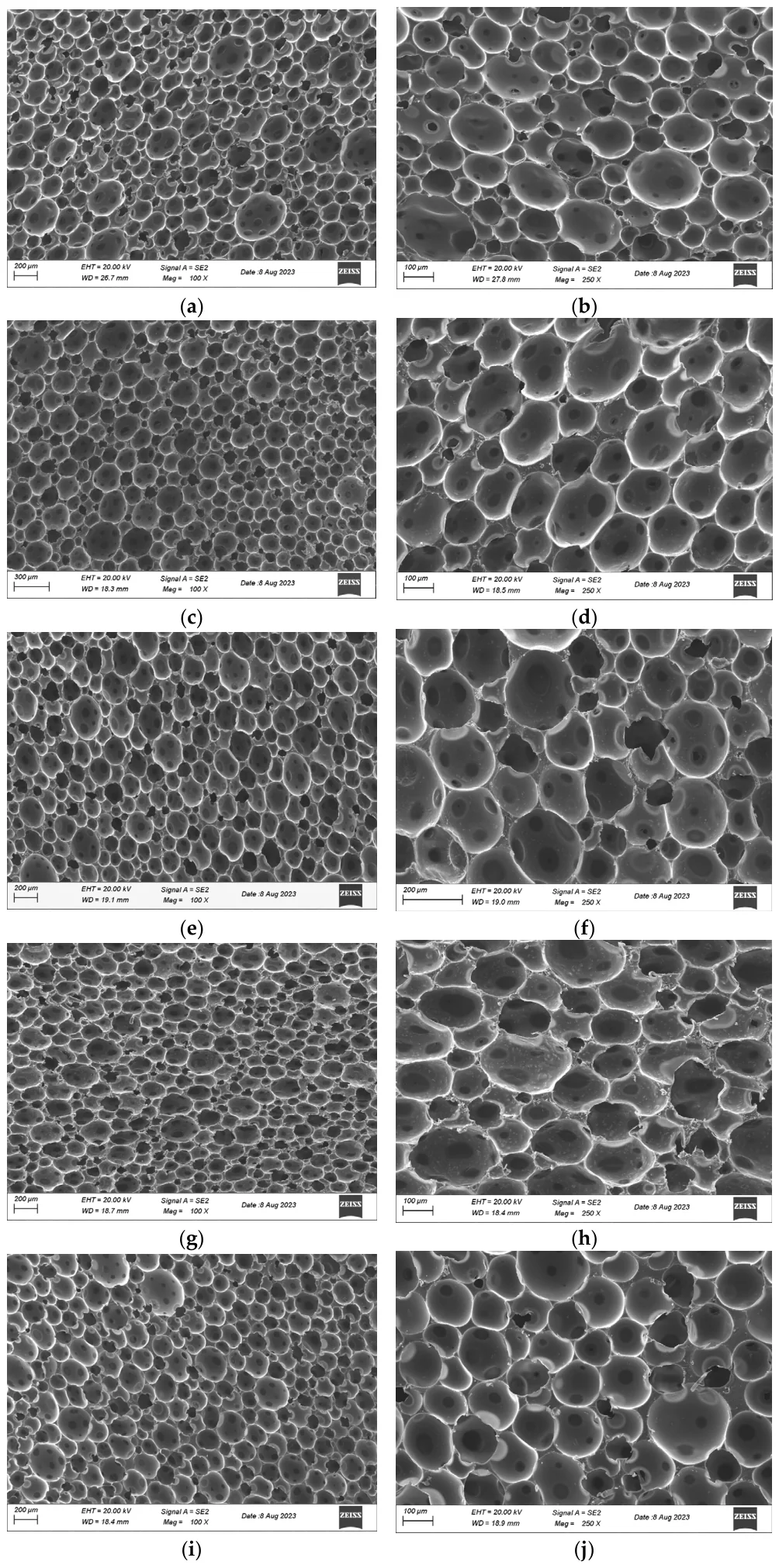
SEM micrographs of PU composites with different pumice contents: (**a**,**b**) PU-00; (**c**,**d**) PU-02; (**e**,**f**) PU-05; (**g**,**h**) PU-10; and (**i**,**j**) PU-20. Images were obtained at magnifications of 100× and 250×, respectively.

The effect of pumice content on the microstructure of the PU composites was examined by SEM. Representative micrographs of the unfilled PU and composites containing 2, 5, 10, and 20 wt.% pumice are presented in Figure 1 at magnifications of 100× and 250×. Some localized tearing and deformation of the cell walls, which are visible in Figure 1, particularly at higher magnification, may represent specimen-preparation artifacts introduced during cutting prior to SEM observation.

The unfilled PU exhibited a relatively regular cellular morphology without prominent cracks or large structural defects (Figure 1a,b). The composite containing 2 wt.% pumice retained a generally similar morphology, with no pronounced particle clustering or interfacial gaps apparent at the examined magnifications (Figure 1c,d). At 5 wt.% pumice, the surface became slightly rougher, with localized irregularities and small pits, while the overall continuity of the PU matrix was maintained (Figure 1e,f).

Further increasing the pumice content to 10 and 20 wt.% resulted in somewhat greater local roughness and heterogeneity (Figure 1g–j). Shallow surface irregularities and uneven topographical features became more evident, particularly at the higher filler contents. Nevertheless, no abrupt or systematic morphological transition was observed over the investigated filler range. The overall matrix morphology remained comparable among the formulations, although the localized heterogeneity tended to become more pronounced as the pumice content increased.

These observations indicate that pumice could be incorporated into the PU matrix over the investigated composition range without producing extensive macroscopic defects. At the same time, the increasing local heterogeneity at higher filler contents may influence stress distribution and matrix continuity. This microstructural response is considered in conjunction with the tensile, abrasion, and cyclic flexural results in the following sections to clarify the relationship between filler content and mechanical performance. The relatively limited morphological change at low pumice content and the greater local heterogeneity observed at higher filler levels are consistent with the filler-content-dependent behavior reported for other mineral-filled flexible PU foams. Javni et al. [14] found that micro-silica and micro-clay could be incorporated over a relatively broad concentration range while maintaining a generally regular cellular structure, whereas greater morphological disruption was observed for some nanoclay-containing foams. Although the filler type and particle characteristics differ from those used in the present study, these findings support the observation that the extent of morphological modification in flexible PU foams depends on both filler characteristics and concentration.

Energy-dispersive X-ray (EDX) spectroscopy was used to evaluate the elemental composition of the unfilled PU and pumice-containing composites, and the results are summarized in Table 2. The unfilled PU was predominantly characterized by carbon and oxygen, whereas Si and Al were detected after pumice incorporation, consistent with the aluminosilicate-rich composition of the mineral filler. As the pumice content increased from 2 to 20 wt.%, the relative contributions of Si and Al generally increased, accompanied by a corresponding decrease in the relative contribution of the polymer-associated elements.

The progressive increase in the inorganic elemental contribution with filler loading provides compositional evidence for the incorporation of pumice into the PU composites. These results complement the SEM observations and confirm the increasing presence of the mineral phase as the pumice content was increased.

**Figure 2:**
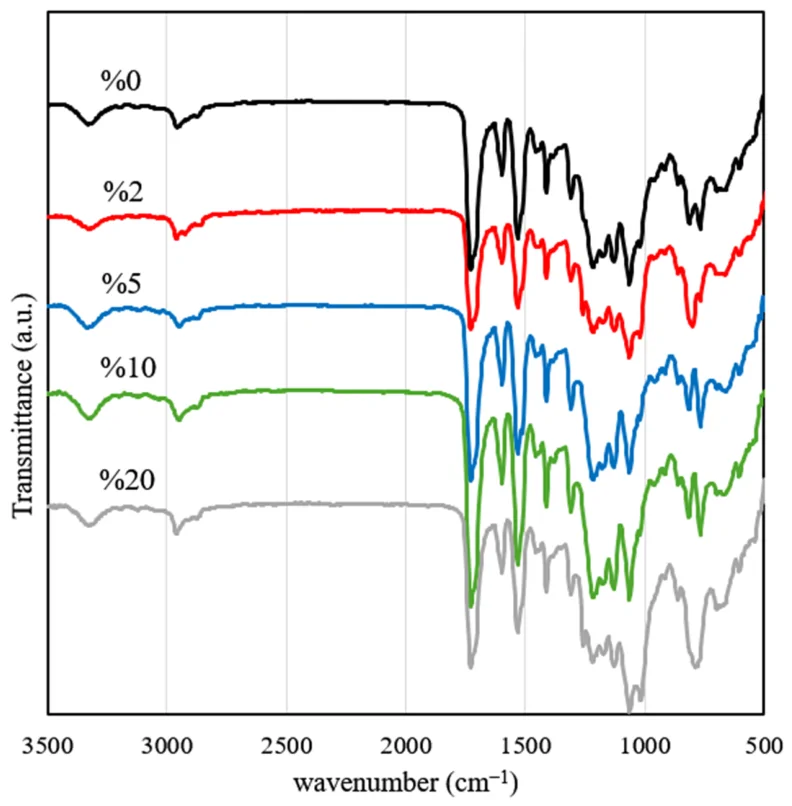
FTIR spectra of the unfilled PU and PU composites containing 2, 5, 10, and 20 wt.% pumice.

FTIR spectra of the unfilled PU and PU composites containing 2, 5, 10, and 20 wt.% pumice are presented in Figure 2. All formulations exhibited the characteristic absorption bands of the PU matrix. The broad band at approximately 3320 cm^−1^ was assigned to N–H stretching, while the bands between 2850 and 2950 cm^−1^ corresponded to the stretching vibrations of aliphatic C–H groups. The prominent band near 1725 cm^−1^ was attributed to the C=O stretching vibration of urethane carbonyl groups. These assignments are consistent with those reported by Dumont and Narine [33], who identified the N–H and C=O vibrational regions as characteristic of urethane linkages in PU foams. Additional characteristic bands were observed at approximately 1595 cm^−1^ and 1220–1240 cm^−1^, while the band around 1100 cm^−1^ was assigned to C–O–C stretching.

The overall spectral profiles remained comparable over the investigated pumice-content range, and no distinct new absorption bands appeared following pumice incorporation. Only relatively small variations in the intensities of some characteristic PU bands were observed with increasing filler content. This behavior contrasts with the results reported by Nordi et al. [34] for jute cellulose nanofiber-reinforced TPU composites, where filler incorporation produced more noticeable changes in the intensities of several characteristic FTIR bands, particularly those associated with O–H, C–H, N–H, and C=O groups. These differences may be associated with the different chemical nature of the fillers and their interactions with the respective PU matrices. In the present system, the absence of distinct new bands and the preservation of the principal PU absorption features indicate that pumice incorporation did not produce pronounced changes in the characteristic FTIR profile of the PU matrix within the resolution of the analysis. Accordingly, the differences in mechanical performance among the formulations are considered primarily in relation to filler content and the associated microstructural effects rather than major changes in the principal chemical structure of the PU matrix.

### 3.2. Physical Properties

**Figure 3:**
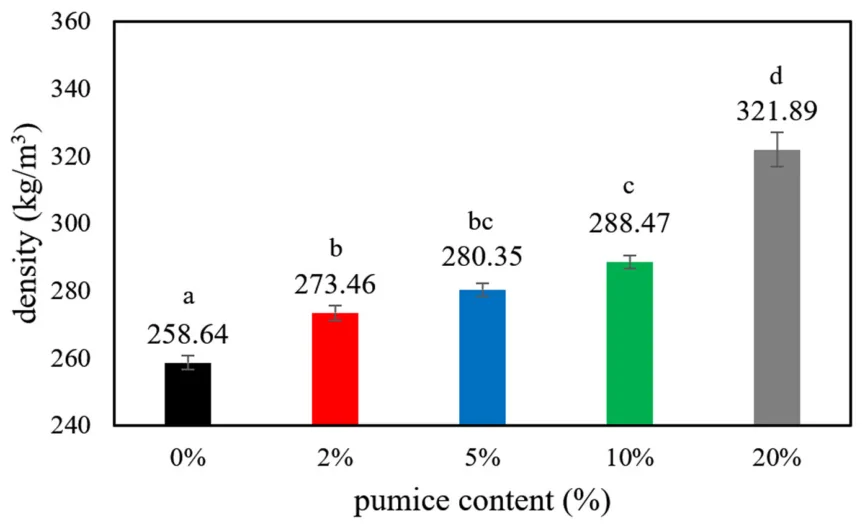
Density of the unfilled PU and PU composites containing different pumice filler contents. Values are presented as mean ± SD (*n* = 3). Different letters indicate statistically significant differences according to Tukey’s HSD test (*p* < 0.05).

The density of the PU composites increased progressively with increasing pumice content, as shown in Figure 3. The unfilled PU exhibited the lowest density of 258.64 kg/m^3^. With the addition of 2 and 5 wt.% pumice, the density increased to 273.46 and 280.35 kg/m^3^, respectively. Further increases in filler content resulted in densities of 288.47 kg/m^3^ at 10 wt.% and 321.89 kg/m^3^ at 20 wt.% pumice. Thus, compared with the unfilled PU, the density increased by approximately 5.7, 8.4, 11.5, and 24.5% for the composites containing 2, 5, 10, and 20 wt.% pumice, respectively. One-way ANOVA confirmed a significant effect of pumice content on composite density (*p* < 0.0001). Tukey’s HSD test showed significant differences among most formulations, whereas the difference between the 2 and 5 wt.% composites was not statistically significant (*p* > 0.05).

The systematic increase in density is consistent with the increasing proportion of the inorganic mineral phase in the PU composites. A similar tendency has been reported for other mineral-filled flexible PU systems. Javni et al. [14] observed moderate increases in foam density with increasing micro-silica and micro-clay contents. In contrast, an earlier study on micro- and nanosilica-filled PU foams [35] reported comparatively limited changes in reduced density over the investigated filler range. These findings indicate that the effect of filler addition on PU foam density is not universal but depends on filler characteristics, concentration, and their influence on the cellular structure. The relatively moderate density changes up to 10 wt.% pumice, followed by a more pronounced increase at 20 wt.%, indicating that filler loading becomes increasingly influential on the bulk physical characteristics of the composite at higher concentrations.

This trend is also consistent with the EDX results, which showed an increasing contribution of the pumice-associated inorganic constituents with increasing filler content.

The density increase represents an important consideration in evaluating the overall effect of pumice incorporation. Although pumice is generally regarded as a lightweight mineral filler, increasing its content resulted in a measurable increase in composite density relative to the unfilled PU. Therefore, the mechanical performance changes discussed in the following sections should be considered together with the corresponding increase in composite density when assessing the overall effect of pumice incorporation.

**Figure 4:**
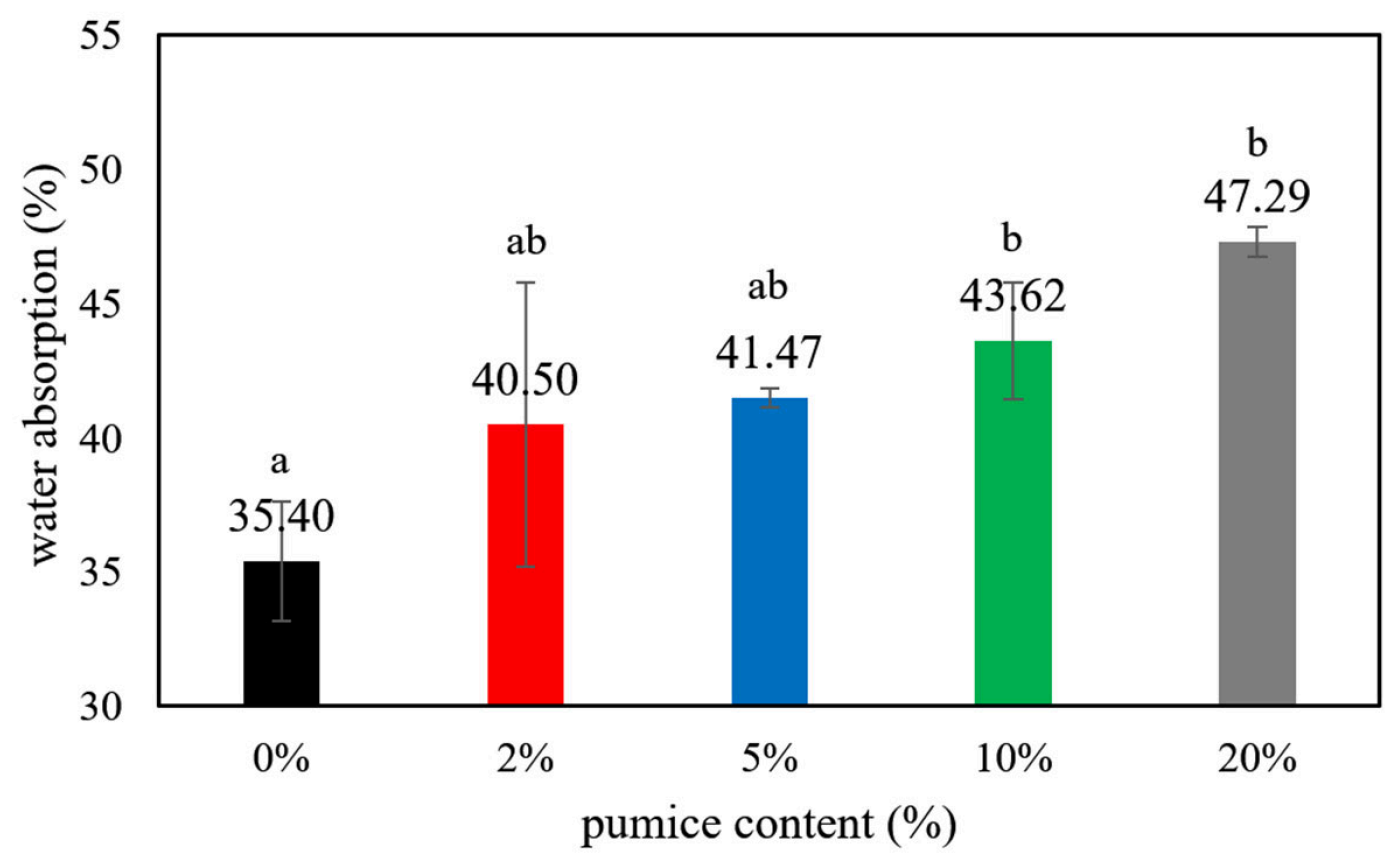
Water absorption of the unfilled PU and PU composites containing different pumice filler contents. Values are presented as mean ± SD (*n* = 3). Different letters indicate statistically significant differences according to Tukey’s HSD test (*p* < 0.05).

Water absorption increased progressively with increasing pumice content, as shown in Figure 4. The unfilled PU exhibited the lowest water absorption of 35.40%. The incorporation of 2 and 5 wt.% pumice increased the water uptake to 40.50 and 41.47%, respectively, while further increases in filler content resulted in values of 43.62% at 10 wt.% and 47.29% at 20 wt.% pumice. Relative to the unfilled PU, these values correspond to increases in water absorption of approximately 14.4, 17.2, 23.2, and 33.6% for the composites containing 2, 5, 10, and 20 wt.% pumice, respectively. One-way ANOVA showed a significant overall effect of pumice content on water absorption (*p* = 0.0046). Tukey’s HSD test indicated significantly higher water absorption for the 10 and 20 wt.% composites compared with the unfilled PU (*p* < 0.05), whereas the differences involving the 2 and 5 wt.% composites were not statistically significant.

The observed trend indicates that increasing the proportion of the mineral phase promotes greater water uptake in the PU composites. Pumice has a porous structure, and its incorporation introduces additional mineral surfaces capable of interacting with water. In addition, the increasing local surface irregularity and heterogeneity observed by SEM at higher filler contents may contribute to greater accessibility and retention of water within the composite. A comparable increase in water uptake following filler incorporation has been reported for other filled PU foams. Afonso et al. [36] found that polyurethane foams containing porous biofillers exhibited substantially higher water absorption than the unfilled PU, with the increase depending on the type of filler. Although the filler chemistry differs from that of pumice, their results similarly demonstrate that the incorporation of a porous dispersed phase can substantially modify the water uptake behavior of PU foams. However, because pore-size distribution and filler–matrix interfacial voids were not independently quantified in the present study, the increase in water absorption cannot be attributed to a specific transport mechanism.

The increase in water absorption represents a relevant trade-off associated with increasing pumice content, particularly for applications in which the material may be exposed to humid or wet service conditions. The relatively moderate change between 2 and 5 wt.% pumice was followed by a further increase at higher filler contents, with the maximum water uptake observed at 20 wt.%. These results should therefore be considered together with the mechanical and durability responses when assessing the overall influence of pumice content on composite performance.

### 3.3. Tensile Behavior

The tensile strength and elongation at break of the unfilled PU and pumice-containing composites are summarized in Table 3. The unfilled PU exhibited a mean tensile strength of 1.90 ± 0.15 MPa and an elongation at break of 201.24 ± 14.42%. The incorporation of 2 wt.% pumice resulted in corresponding values of 1.85 ± 0.06 MPa and 157.24 ± 42.64%, respectively. The highest mean tensile strength was obtained for the 5 wt.% composite (2.11 ± 0.10 MPa), followed by the 10 wt.% composite (2.04 ± 0.34 MPa), whereas the 20 wt.% composite exhibited a mean tensile strength of 1.82 ± 0.07 MPa.

Despite these numerical differences, one-way ANOVA showed that the effect of pumice content on tensile strength was not statistically significant (*p* = 0.272). Therefore, the higher mean tensile strengths observed at 5 and 10 wt.% pumice should be interpreted as a tendency rather than a statistically demonstrated reinforcement effect. In contrast, elongation at break decreased progressively with increasing pumice content, from 201.24 ± 14.42% for the unfilled PU to 122.17 ± 2.51% for the 20 wt.% composite. The overall effect of pumice content on elongation was statistically significant (*p* = 0.0196), and Tukey’s HSD test identified a significant difference between the unfilled PU and the 20 wt.% composite (*p* = 0.0121).

These results indicate that, within the investigated composition range, pumice incorporation had a more pronounced effect on deformability than on tensile strength. The progressive reduction in elongation suggests increasing restriction of matrix deformation as the mineral fraction increased. The greater local heterogeneity observed by SEM at higher pumice contents may also contribute to less uniform deformation. However, since interfacial adhesion and particle agglomeration were not independently quantified, the observed tensile response cannot be attributed exclusively to a specific microstructural mechanism.

The present behavior is generally consistent with the filler-content-dependent response reported for other filled PU systems. Javni et al. [14] reported that mineral microfillers had only a limited effect on tensile strength while reducing elongation at break. Similarly, Husainie et al. [23] observed that the tensile response of natural-filler-containing PU foams depended on filler loading. Although the filler types and PU formulations differ from those used in the present study, these findings support the observation that filler incorporation may affect deformability more clearly than tensile strength and that increasing filler content does not necessarily result in continuous tensile reinforcement.

### 3.4. Abrasion Resistance

**Figure 5:**
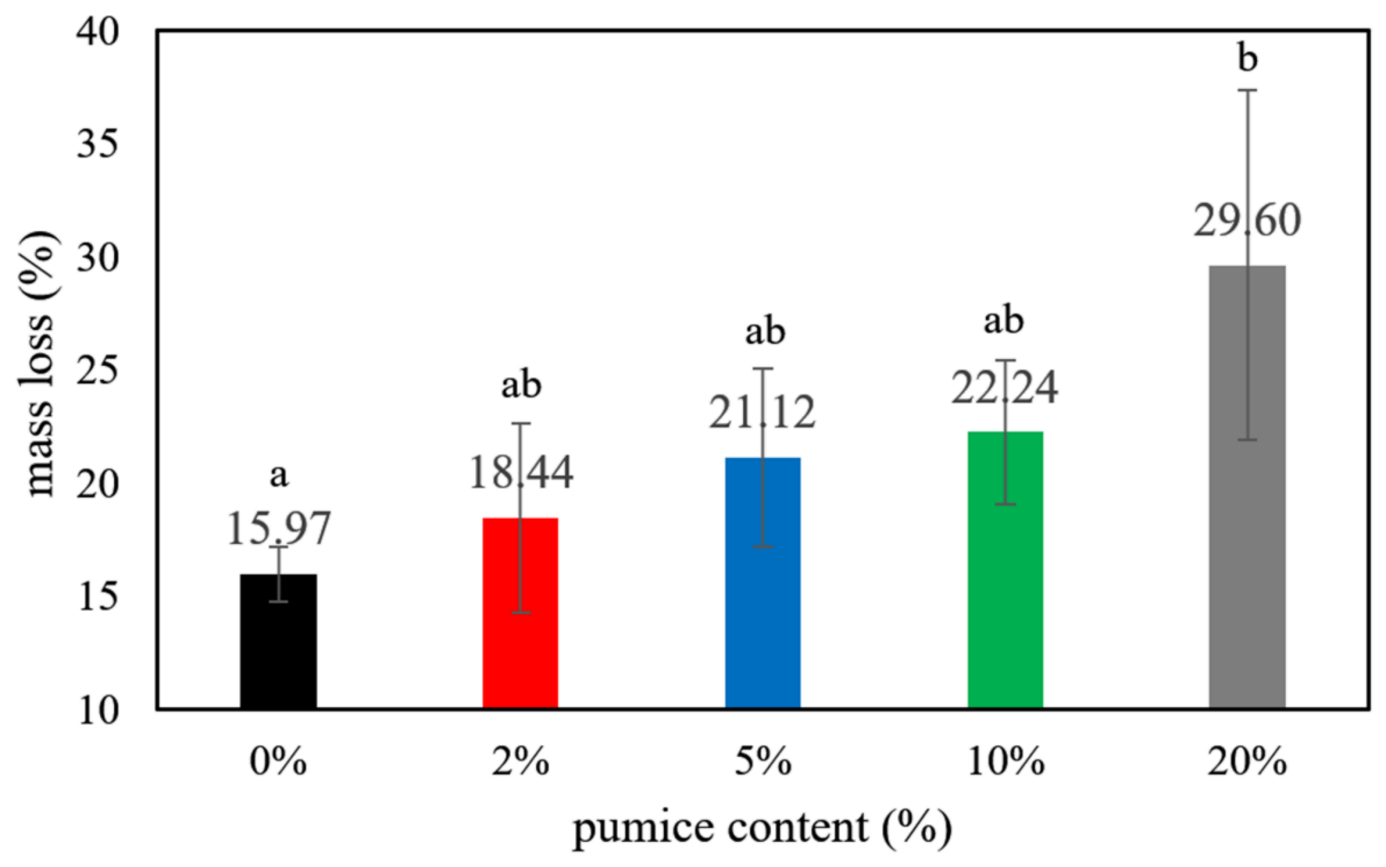
Average mass loss of the unfilled PU and PU composites containing different pumice filler contents after abrasion testing. Values are presented as mean ± SD (*n* = 3). Different letters indicate statistically significant differences according to Tukey’s HSD test (*p* < 0.05).

Abrasion resistance was evaluated based on the percentage mass loss after testing, with lower mass loss corresponding to higher resistance to abrasive wear. As shown in Figure 5, the unfilled PU exhibited the lowest mass loss of 15.97%. The incorporation of 2 wt.% of pumice increased the mass loss to 18.44%, corresponding to a reduction in abrasion resistance. Further increases in pumice content resulted in mass losses of 21.12 and 22.24% for the 5 and 10 wt.% composites, respectively, while the highest mass loss of 29.60% was obtained at 20 wt.% pumice.

Relative to the unfilled PU, the mass loss increased by approximately 15.5, 32.2, 39.3, and 85.3% for the composites containing 2, 5, 10, and 20 wt.% pumice, respectively. One-way ANOVA indicated a significant overall effect of pumice content on abrasion mass loss (*p* = 0.0388). However, Tukey’s HSD test identified a statistically significant difference only between the unfilled PU and the 20 wt.% composite (*p* < 0.05), while the differences among the other formulations were not statistically significant. The mean values therefore show a progressive deterioration in abrasion resistance with increasing filler content. The relatively small difference between the 5 and 10 wt.% formulations further indicates that the somewhat higher mean tensile strengths observed at intermediate filler contents did not translate into a corresponding improvement in resistance to abrasive material removal. This distinction highlights the property-dependent role of pumice in the mechanical response of the composites.

The reduction in abrasion resistance may be related to the increasing proportion of rigid mineral particles within the flexible PU matrix. Under repeated abrasive contact, local differences in deformation between the polymer matrix and the dispersed mineral phase may facilitate localized material removal. At higher filler contents, the greater local surface roughness and heterogeneity observed by SEM may further contribute to the increased susceptibility to abrasive wear. However, since particle pull-out and interfacial failure were not directly characterized after abrasion testing, their individual contributions to the measured mass loss cannot be conclusively distinguished.

Overall, the abrasion results demonstrate that increasing pumice content adversely affects the wear resistance of the flexible PU composites within the investigated composition range. This behavior differs from that reported for some other mineral-filled PU systems. Shahzamani et al. [19] showed that the abrasion response of cast PU varied considerably with filler type and content, with CaCO_3_- and BaSO_4_-containing formulations exhibiting lower abrasion volume loss than quartz-containing formulations. They also associated poorer abrasion resistance in some filler systems with filler distribution and increased surface roughness. Although the PU system, filler type, loading basis, and abrasion metric differ from those used in the present study, these results demonstrate that mineral filler incorporation does not have a universally beneficial or detrimental effect on abrasion resistance. In the present pumice-filled system, the progressive increase in mean mass loss indicates that the effects associated with increasing mineral content and microstructural heterogeneity were accompanied by a deterioration in abrasion resistance, despite the somewhat higher mean tensile strengths observed at intermediate filler contents. This trade-off is particularly relevant for applications involving repeated surface contact and friction.

### 3.5. Cyclic Flexural Durability at Different Service Temperatures

The cyclic flexural durability of the PU composites was evaluated at 20, 0, and −20 °C to determine the combined influence of pumice content and service temperature on resistance to crack propagation under repeated deformation. The results after 30,000 flexing cycles are summarized in Table 4. A clear dependence on both filler content and test temperature was observed.

At 20 °C, the unfilled PU and the composite containing 2 wt.% pumice exhibited the highest resistance to cyclic crack propagation. In both formulations, the initial 2 mm notch remained unchanged after testing, indicating that the specimens withstood the imposed cyclic deformation without measurable crack extension. Increasing the pumice content to 5 wt.% markedly reduced cyclic durability, with the notch extending from 2 to 40 mm, corresponding to a crack growth of 1900%. At higher filler contents of 10 and 20 wt.%, complete fracture occurred during the test. Thus, at 20 °C, a distinct deterioration in cyclic flexural resistance became evident when the pumice content reached 5 wt.% and above.

The influence of filler content became more pronounced at 0 °C. The unfilled PU and the 2 wt.% composite again completed the test without measurable crack growth, whereas all composites containing 5 wt.% or more pumice underwent complete fracture. The transition from severe crack propagation at 20 °C for the 5 wt.% composite to complete fracture at 0 °C demonstrates that the resistance of the filled composites to repeated flexural deformation becomes increasingly sensitive to filler content as the service temperature decreases.

At −20 °C, all formulations, including the unfilled PU, underwent complete fracture. Under these conditions, the dominant influence of the low test temperature prevented differentiation among the investigated filler contents based on the final failure state. The complete fracture of even the unfilled PU indicates that the cyclic flexural resistance of the base matrix itself was insufficient under the imposed test conditions at −20 °C.

Taken together, the results demonstrate that cyclic mechanical durability is strongly dependent on both pumice content and service temperature. At 20 and 0 °C, the unfilled PU and the 2 wt.% composite exhibited substantially better resistance to crack propagation than formulations containing higher pumice contents. In contrast, the 5 wt.% formulation showed a temperature-dependent transition from severe crack growth at 20 °C to complete fracture at 0 °C, while the 10 and 20 wt.% formulations completely fractured at both temperatures. These findings indicate that increasing pumice content progressively limits the ability of the flexible PU composite to withstand repeated deformation, with the adverse effect becoming more critical under lower-temperature service conditions.

This behavior also demonstrates an important property trade-off within the investigated composition range. Although the 5 and 10 wt.% formulations exhibited the highest mean tensile strengths, these differences were not statistically significant and were not accompanied by enhanced cyclic flexural durability. Instead, the resistance to repeated deformation deteriorated substantially at filler contents of 5 wt.% and above. The sensitivity of flexible PU materials to repeated loading has also been demonstrated by Foster et al. [37], who reported that the cyclic mechanical response of open-cell flexible PU foams depends on material formulation and testing conditions, including loading amplitude and frequency. Although their study did not investigate mineral-filled PU composites or low-temperature flexural crack propagation, it demonstrates that the cyclic response of flexible PU cannot be inferred directly from monotonic mechanical properties. In the present study, the additional dependence on pumice content and test temperature further shows that tensile performance alone is insufficient for assessing resistance to repeated deformation.

From a practical material-selection perspective, pumice incorporation presents both benefits and limitations. Intermediate filler contents, particularly 5 and 10 wt.%, produced somewhat higher mean tensile strength, although the differences among formulations were not statistically significant, while the 2 wt.% composite retained the cyclic flexural durability of the unfilled PU at 20 and 0 °C. On the other hand, increasing pumice content leads to higher density and water absorption and progressively reduces abrasion resistance, while cyclic flexural durability deteriorates at filler contents of 5 wt.% and above. These competing effects indicate that the filler content should be selected according to the dominant performance requirements rather than on the basis of tensile performance alone.

## 4. Conclusions

The present study demonstrates that the effect of natural pumice on flexible polyester-based PU composites depends strongly on filler content and on the properties considered. Pumice incorporation produced a progressive increase in density and water absorption at higher filler contents. The 5 and 10 wt.% pumice-added samples exhibited higher mean tensile strengths than the unfilled PU, although the differences in tensile strength among the samples were not statistically significant. In contrast, elongation at break decreased significantly with increasing pumice content, indicating that the main effect of the mineral filler on tensile behavior was a reduction in deformability rather than an increase in strength. Abrasion resistance also deteriorated as the filler content increased, with the greatest mass loss observed at 20 wt.% pumice.

Cyclic flexural behavior provided a clearer distinction among the samples with different pumice content. The 2 wt.% pumice-added composite completed 30,000 cycles at both 20 and 0 °C without measurable crack propagation, showing cyclic flexural behavior comparable to that of the unfilled PU under these conditions. This behavior was not maintained at higher filler contents. The 5 wt.% composite showed severe crack growth at 20 °C and complete fracture at 0 °C, while the 10 and 20 wt.% composites fractured completely at both temperatures. At −20 °C, complete fracture occurred in all samples, including the unfilled PU. These results show that cyclic durability is governed by the combined effects of filler content and service temperature and cannot be predicted from tensile strength alone.

Taken together, the findings do not indicate a single pumice content that simultaneously improves all of the investigated properties. Low pumice loading, particularly 2 wt.%, appears more suitable when retention of cyclic flexural durability is required, whereas increasing the filler content introduces penalties in deformability, abrasion resistance, water uptake, and density without providing a statistically significant increase in tensile strength. Pumice can therefore be used as a natural mineral filler in flexible PU composites, but its content should be selected according to the intended service conditions and the relative importance of the required properties. Further work should focus on filler–matrix interfacial characteristics, particle dispersion, and approaches to improving low-temperature cyclic durability and abrasion resistance in pumice-containing flexible PU composites.

## Figures and Tables

**Table 1:** Composition of PU composites with varying pumice content.

Sample Code	Content (wt.%)		
	Pumice (C)	Polyol Blend (A)	Isocyanate Component (B)
PU-00	0	50	50
PU-02	2	49	49
PU-05	5	47.5	47.5
PU-10	10	45	45
PU-20	20	40	40

**Table 2:** Elemental composition of the unfilled PU and pumice-containing PU composites determined by EDX analysis.

Code	Polymer (%)	Si (%)	Al (%)	Others (%)
PU-00	99.21	0.75	-	0.04
PU-02	98.49	1.30	-	0.21
PU-05	94.18	4.66	0.20	0.96
PU-10	88.62	10.37	0.90	0.11
PU-20	80.69	15.40	1.40	2.51

**Table 3:** Tensile properties of the unfilled PU and PU composites containing different pumice filler contents.

Code	Pumice Content (wt.%)	Tensile Strength (MPa)	Elongation at Break (%)
PU-00	0	1.90 ± 0.15 ^a^	201.24 ± 14.42 ^a^
PU-02	2	1.85 ± 0.06 ^a^	157.24 ± 42.64 ^ab^
PU-05	5	2.11 ± 0.10 ^a^	148.22 ± 9.69 ^ab^
PU-10	10	2.04 ± 0.34 ^a^	142.66 ± 22.74 ^ab^
PU-20	20	1.82 ± 0.07 ^a^	122.17 ± 2.51 ^b^

Values are presented as mean ± standard deviation (*n* = 3). Different superscript letters within the same column indicate statistically significant differences according to Tukey’s HSD test (*p* < 0.05). One-way ANOVA indicated no significant effect of pumice content on tensile strength (*p* = 0.272), whereas the effect on elongation at break was significant (*p* = 0.0196).

**Table 4:** Cyclic flexural durability of the unfilled PU and pumice-containing composites at different test temperatures.

Temp.	Sample Code	Final Notch Length (mm)	Crack Growth (%)	Failure Type
20 °C	PU-00	2	0	No Failure
	PU-02	2	0	No Failure
	PU-05	40	1900	Severe Crack Growth
	PU-10	CF	CF	CF
	PU-20	CF	CF	CF
0 °C	PU-00	2	0	No Failure
	PU-02	2	0	No Failure
	PU-05	CF	CF	CF
	PU-10	CF	CF	CF
	PU-20	CF	CF	CF
−20 °C	PU-00	CF	CF	CF
	PU-02	CF	CF	CF
	PU-05	CF	CF	CF
	PU-10	CF	CF	CF
	PU-20	CF	CF	CF

Initial notch length: 2 mm; CF: complete fracture.

## Data Availability

The data presented in this study are available upon request from the corresponding author.
